# Constrained spherical deconvolution-based tractography and tract-based spatial statistics show abnormal microstructural organization in Asperger syndrome

**DOI:** 10.1186/2040-2392-6-4

**Published:** 2015-01-16

**Authors:** Ulrika Roine, Juha Salmi, Timo Roine, Taina Nieminen-von Wendt, Sami Leppämäki, Pertti Rintahaka, Pekka Tani, Alexander Leemans, Mikko Sams

**Affiliations:** Brain and Mind Laboratory, Department of Biomedical Engineering and Computational Science, Aalto University, Rakentajanaukio 2 C, FI-02150 Espoo, Finland; iMinds-Vision Lab, Department of Physics, University of Antwerp, Universiteitsplein 1, B-2610 Wilrijk, Antwerp Belgium; Neuropsychiatric Rehabilitation and Medical Centre Neuromental, Kaupintie 11 A, FI-00440 Helsinki, Finland; Department of Psychiatry, Clinic for Neuropsychiatry, Helsinki University Central Hospital, Tukholmankatu 8 F, FI-00290 Helsinki, Finland; Finnish Institute of Occupational Health, Topeliuksenkatu 41, FI-00290 Helsinki, Finland; Image Sciences Institute, University Medical Center Utrecht, Heidelberglaan 100, 3584 CX Utrecht, The Netherlands; Advanced Magnetic Imaging Centre, Aalto University, Otakaari 5, FI-02150 Espoo, Finland

**Keywords:** autism spectrum disorder, diffusion magnetic resonance imaging, fractional anisotropy, white matter tract, inferior longitudinal fasciculus

## Abstract

**Background:**

The aim of this study was to investigate potential differences in neural structure in individuals with Asperger syndrome (AS), high-functioning individuals with autism spectrum disorder (ASD). The main symptoms of AS are severe impairments in social interactions and restricted or repetitive patterns of behaviors, interests or activities.

**Methods:**

Diffusion weighted magnetic resonance imaging data were acquired for 14 adult males with AS and 19 age, sex and IQ-matched controls. Voxelwise group differences in fractional anisotropy (FA) were studied with tract-based spatial statistics (TBSS). Based on the results of TBSS, a tract-level comparison was performed with constrained spherical deconvolution (CSD)-based tractography, which is able to detect complex (for example, crossing) fiber configurations. In addition, to investigate the relationship between the microstructural changes and the severity of symptoms, we looked for correlations between FA and the Autism Spectrum Quotient (AQ), Empathy Quotient and Systemizing Quotient.

**Results:**

TBSS revealed widely distributed local increases in FA bilaterally in individuals with AS, most prominent in the temporal part of the superior longitudinal fasciculus, corticospinal tract, splenium of corpus callosum, anterior thalamic radiation, inferior fronto-occipital fasciculus (IFO), posterior thalamic radiation, uncinate fasciculus and inferior longitudinal fasciculus (ILF). CSD-based tractography also showed increases in the FA in multiple tracts. However, only the difference in the left ILF was significant after a Bonferroni correction. These results were not explained by the complexity of microstructural organization, measured using the planar diffusion coefficient. In addition, we found a correlation between AQ and FA in the right IFO in the whole group.

**Conclusions:**

Our results suggest that there are local and tract-level abnormalities in white matter (WM) microstructure in our homogenous and carefully characterized group of adults with AS, most prominent in the left ILF.

## Background

Asperger syndrome (AS) is a neurodevelopmental autism spectrum disorder (ASD), which affects 0.6 to 0.7% of the population [1]. The main symptoms of AS are severe impairments in social interactions and restricted, repetitive patterns of behaviors, interests, and activities. Although the heritability of ASD has been estimated to be as high as 90% [2], no single autism locus has been implicated. Rather, autism seems to be a behavioral manifestation of multiple underlying genetic disorders [3]. This heterogeneity, the diversity of symptoms, and the variation in the degree of their severity among individuals, also suggest that the neurobiological background is very complex with multiple brain areas being involved. It has been suggested that there are widely distributed abnormalities in brain connectivity in individuals with ASD [4–6].

Functional imaging studies have shown that there is reduced long-distance and increased short-distance connectivity in ASD [4–6]. Recently, diffusion-weighted (DW) magnetic resonance imaging (MRI) has enabled also the *in vivo* investigation of anatomical brain connectivity [7–10]. In white matter (WM) tracts, the diffusion of water molecules is hindered more by the cell walls of the axons than along the main orientation of the axons, and thus, diffusion is likely to be anisotropic in WM. Fractional anisotropy (FA) is the most commonly used index to describe the degree of anisotropy and can be used to quantify the microstructural coherence or organization of WM tracts [11]. Mean diffusivity (MD) represents the average diffusion rate in all directions and with the planar diffusion coefficient (CP), the degree of fiber complexity can be quantified [12]. In particular, a higher CP describes a more disc-shaped diffusion, typically caused by ‘crossing fibers’ [13–15].

The findings of diffusion MRI studies in ASD are not consistent. Both decreased and increased FA values have been reported in many WM tracts in ASD, compared to typically developing control subjects [16, 17]. Most of the studies have been performed in either children or adolescents with ASD [17]. In adults, mainly decreased FA values in ASD subjects have been reported [18–23]. Bloemen and coworkers [20] used a voxelwise method to analyze the FA values of the WM in 13 adult males with AS and 13 age and IQ-matched male controls and mainly found regions with decreased FA in adults with AS. The discrepancy across studies could be partly due to the use of different image acquisition parameters or analysis approaches [17, 24–26], but also the variation in age and cognitive profile of the subjects across the different studies may play a role in this context.

The aim of our study was to investigate potential differences in FA and MD in adults with ASD. To narrow down the variation in the cognitive profile of the subjects, we only investigated high-functioning individuals with AS. Individuals with AS and autism share the same core symptoms, but according to DSM-IV, subjects with AS do not have a clinically significant delay in speech and cognitive development, although in DSM-5, autism and AS (among others) were placed on the same spectrum of autistic disorders.

As different analytic approaches may also contribute to the varying results in DWI studies in ASD, we have chosen to use multiple approaches to confirm that the results support each other. In our previous histogram-based study, we used a dual approach, a skeletonized white matter histogram and a whole-brain tractography histogram, and found a global increase of FA values in adult males with AS compared to age-, sex- and IQ-matched controls, suggesting a more coherent neural tract organization in the individuals with AS [27]. This raised a question about the location of the differences. Thus, in the current study, the aim was to find out if the differences would be widely distributed or if they could be pointed more specifically to certain locations or white matter tracts. We chose again a dual approach and used both tract-based spatial statistics (TBSS) [28] and tractography to investigate both voxelwise differences and tract-level differences. Furthermore, as diffusion tensor imaging (DTI) is unable to correctly characterize crossing fiber configurations, which are present in up to 90% of the WM tissue [29], we used constrained spherical deconvolution (CSD)-based tractography [30, 31]. In CSD, multiple fibers passing through a voxel with distinct orientations can be reliably estimated. So far, CSD-based tractography has only been used in two studies in ASD [32, 33], and only a few tracts have been investigated.

## Methods

### Participants

Fourteen individuals with AS and 19 control subjects without any neuropsychiatric disorders were included in this study. All subjects were male, and the individuals with AS were age- and IQ-matched with the controls. To minimize the effect of age-related changes on the neural structure, only individuals aged 40 years or less were eligible for the study. The mean age of individuals with AS was 28.6 ± 5.7 years and that of controls 26.4 ± 4.7 years. The mean IQs for the AS and control groups were 125.1 ± 14.5 and 127.9 ± 10.0, respectively (Wechsler’s Adult Intelligence Scale-Third Edition; The Psychological Corporation; 2005). The patients were recruited from a private neuropsychiatric clinic (NeuroMental) in Helsinki and from the neuropsychiatric clinic in Helsinki University Central Hospital. Only individuals fulfilling ICD-10 (International Classification of Disease; World Health Organization; 1993) criteria, diagnosed by experienced clinicians specialized in developmental neuropsychiatry, were included in the study. Both individuals with AS and controls had a full psychiatric evaluation before inclusion in the study. Diagnostic process for the AS group included full developmental history, acquired using multiple sources of information (for example, all previous medical records, parental interviews when possible). Benton Facial Recognition Test (FRT) [34] and Reading the Mind in the Eyes Test (Eyes Test) [35] were carried out for all subjects. In addition, all subjects completed Autism Spectrum Quotient (AQ) [36], Empathy Quotient (EQ) [37] and Systemizing Quotient (SQ) [38] questionnaires, which had been translated into Finnish, and the translation was confirmed by a back-translation. A two-tailed *t*-test was used to test for group differences. There were no significant differences in total IQ, verbal IQ, performance IQ, FRT, Eyes test or SQ, whereas individuals with AS had significantly higher AQ scores (*P* = 0.0000001) and significantly lower EQ scores than controls (*P* = 0.0017) (see Table one in Roine *et al*. [27]). Control subjects were paid for their attendance in the study and for individuals with AS the expenses and the loss of income were compensated. The Ethics Committee of the Hospital District of Helsinki and Uusimaa approved the research protocol, and all participants signed a written informed consent form before participating in the study.

### Image acquisition

The MR images were acquired with a Signa VH/i 3.0 T scanner with HDxt upgrade (General Electric, Milwaukee, WI, USA). A quadrature receiving eight-channel high-resolution brain array coil was used (MRI Devices Corporation, FL). The maximum field gradient amplitude of the MRI system was 40 mT/m with a slew rate of 150 T/m/s. A high-order shimming with 24-cm field of view (FOV) was applied prior to DW imaging. A spin echo pulsed sequence of 60 unique gradient orientations arranged on the unit sphere was used. Eight nondiffusion weighted B0-images were acquired and all of the 60 orientations were imaged twice, resulting in 120 diffusion-weighted images in total. The b-value, which controls the diffusion weighting, was 1000 s/mm^2^. Echo time (TE) was set to the minimum (approximately 98 ms). Repetition time (TR) was 10 s, and the number of excitations (NEX) was one. The imaging area covered the whole brain with 53 contiguous axial slices. The acquired in-plane resolution of the slices was 1.875 mm × 1.875 mm, and the thickness of the slices was 3.0 mm. The matrix size was 128 × 128.

The DW acquisitions of the two groups were tested for possible differences in subject motion using a two-tailed *t*-test. All of the parameters of the affine transform (12 degrees of freedom) were tested both for absolute and relative differences [39]. No group differences were found (*P* < 0.05) in any of the parameters.

### Voxelwise analysis with tract-based spatial statistics

The voxelwise statistical analysis of the diffusion data was carried out using TBSS [28], which belongs to the Functional MRI of the Brain (FMRIB) Software Library (FSL) tools [40]. FMRIB’s Diffusion Toolbox (FDT) was used for the preprocessing of the data. DW images were corrected for subject motion and eddy-current-induced distortions, and the nonbrain tissue was removed. Diffusion tensors were fitted resulting in FA and MD images in addition to the images containing eigenvalues of the diffusion tensor. As differences in FA could result from changes in the fiber complexity [29, 41], CP images were calculated from the eigenvalues [12]. FA images were transformed into standard space by nonlinear registration based on free-form deformations and B-splines [42], after which a mean FA image of all subjects was calculated and thinned, resulting in a mean FA skeleton image. Then FA data of all subjects were projected onto the mean FA skeleton by selecting the highest FA values perpendicular to the FA skeleton. For MD and CP, the same nonlinear warps and projection vectors as used for FA images were used. ‘Randomise’, a permutation program that enables modeling and inference using standard general linear model design setup, was used for statistical testing of the voxelwise differences between the two groups. Threshold-free cluster enhancement (TFCE) was used to enhance cluster-like structures in the data [43]. Permutation-based non-parametric testing was used to correct for multiple comparisons across space. The TBSS results were also transformed back to native space of each subject to confirm that a given point in the skeleton was derived from the correct anatomically corresponding region. We tested for differences in FA, MD and CP values between subjects with AS and controls. In addition, we correlated AQ, SQ and EQ with FA for the whole group including individuals with AS and controls. Analyses were controlled for age and IQ.

### Constrained spherical deconvolution-based tractography

**Figure 1:**
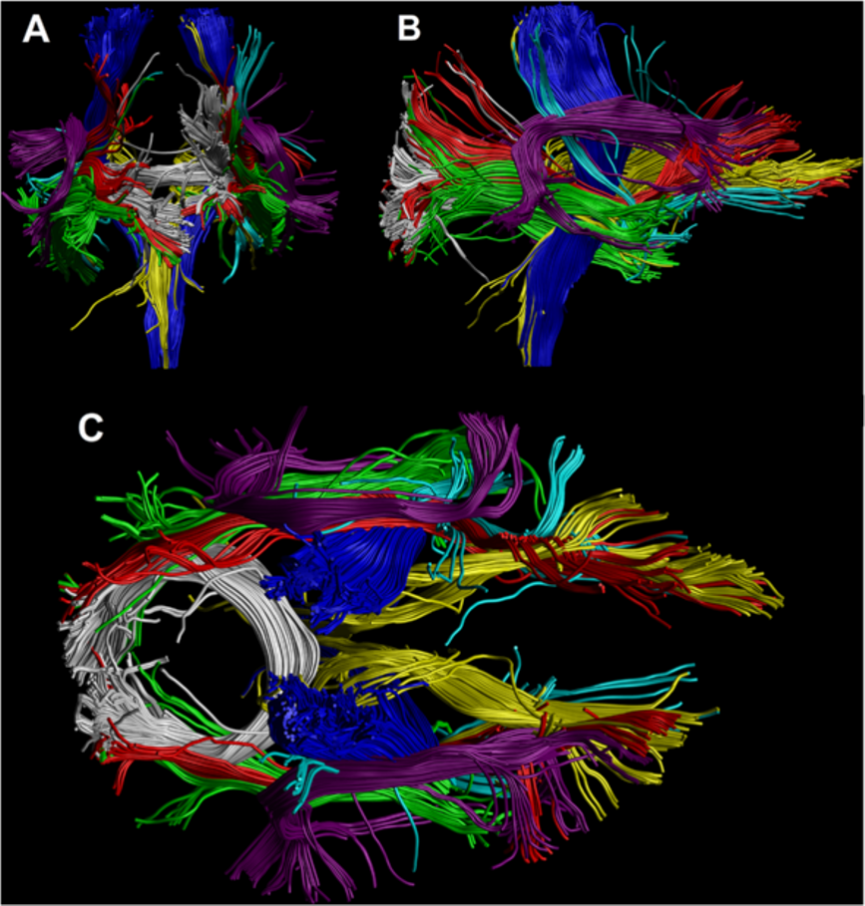
**Coronal (A), sagittal (B) and axial (C) illustrations of the tracts used in the tract-level analysis.** Tracts were extracted with constrained spherical deconvolution (CSD)-based tractography from the diffusion weighted data of a single subject. Corticospinal tract (CST) = blue, splenium of corpus callosum (SCC) = white, anterior thalamic radiation (ATR) = yellow, inferior fronto-occipital fasciculus (IFO) = red, uncinate fasciculus (UNC) = cyan, inferior longitudinal fasciculus (ILF) = green, superior longitudinal fasciculus (SLF) = violet.

CSD-based tractography was performed in ExploreDTI together with the preprocessing of the data [44, 45]. The DW images were corrected for subject motion and eddy-current-induced distortions [46], after which the tensor model was fitted with a nonlinear approach [47]. Then, the fiber orientation distribution functions (FOD) were estimated with CSD, and whole-brain tractography was performed in native space for all subjects [31, 45, 48]. Spherical harmonics up to sixth order were used in the estimation. An FOD threshold of 0.1, a maximum angle deviation of 30 degrees, and a step size of 1 mm was used. The minimum length of the fiber was set to 50 mm. To select the tracts of interest from the whole-brain tractography, regions of interests (ROIs) were defined based on *a priori* information of tract location [49]. The tracts of interest were chosen based on the results in TBSS, and identified with the JHU ICBM-DTI-81 White-Matter Labels Atlas in FSL. A semi-automated method was used to extract 13 tracts, shown in Figure 1, in 33 individuals [50]. The semi-automated method has been validated to produce similar results as manually drawn ROIs and without an increase in the variability of the results [50]. The investigated tracts were splenium of corpus callosum (SCC) and six bilateral tracts: corticospinal tract (CST), anterior thalamic radiation (ATR), inferior fronto-occipital fasciculus (IFO), uncinate fasciculus (UNC), inferior longitudinal fasciculus (ILF), and superior longitudinal fasciculus (SLF). In this procedure, the ROIs for all tracts were selected manually on a color map of one subject, and then transformed to the other subjects’ brains taking into account intersubject variability. In addition, a laterality index (LI) was calculated for all bilateral tracts as follows: LI = (left-right)/(left + right). Two-sample t-tests, assuming unequal variances, were performed to test for group differences in FA, MD, CP and LI between the two groups. Based on earlier results [27] and the results of the TBSS analyses, we used an alternative hypothesis for FA being higher in individuals with AS than in controls. For MD, CP and LI, no prior hypothesis was used. Bonferroni correction for multiple comparisons was used in the tract-level analyses. Correlations between FA and AQ, EQ and SQ scores were calculated both for the whole sample and separately for individuals with AS and controls.

## Results and discussion

A voxelwise comparison of FA, MD and CP values between individuals with AS and age, sex and IQ-matched controls was performed with TBSS, and the findings were confirmed by CSD-based tractography.

### Voxelwise analysis with tract-based spatial statistics

**Figure 2:**
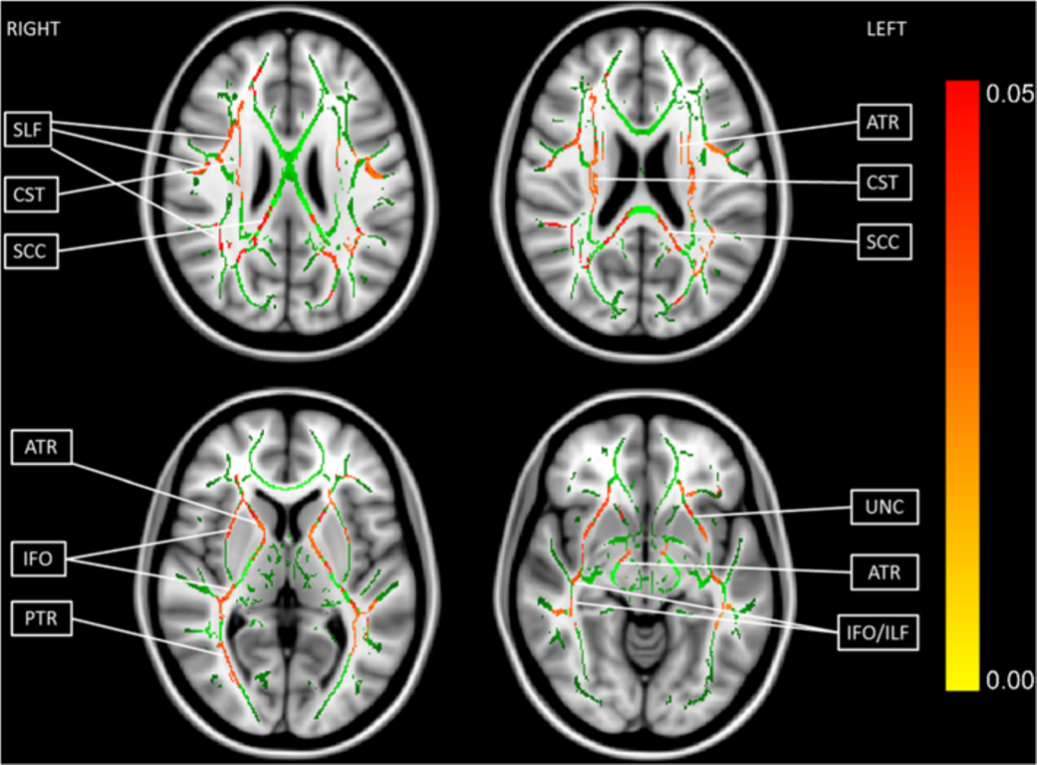
**Tract-based spatial statistics (TBSS) revealed widely distributed local increases in fractional anisotropy (FA) in individuals with Asperger syndrome (AS).** Four axial slices are shown (upper left: slice 97, upper right: slice 93, lower left: slice 77, lower right: slice 64). The green color shows the mean FA skeleton calculated from all subjects by TBSS, and the red color indicates the areas of increased FA in individuals with AS (corrected *P* <0.05). SLF, superior longitudinal fasciculus; CST, corticospinal tract; SCC, splenium of corpus callosum; ATR, anterior thalamic radiation; IFO, inferior fronto-occipital fasciculus; PTR, posterior thalamic radiation; UNC, uncinate fasciculus; ILF, inferior longitudinal fasciculus. The white matter (WM) tracts were identified with the JHU ICBM-DTI-81 White-Matter Labels Atlas in the Functional MRI of the Brain (FMRIB) Software Library (FSL).

Voxelwise comparison with TBSS revealed local bilateral increases in FA in individuals with AS (corrected *P* <0.05) in the following tracts (shown in Figure 2): temporal part of SLF, CST, SCC, ATR, IFO, UNC, ILF and posterior thalamic radiation (PTR). No significant changes were found in MD or CP between the two groups, and no significant correlations were found between AQ, EQ or SQ and FA.

As TBSS is designed for investigation of voxelwise differences in WM tracts, and only the voxels with the highest FA value in the cross-section of the WM tracts are analyzed, the analyses are restricted to WM. This way, the partial volume effect (PVE) is minimized in terms of adverse contributions of GM or cerebrospinal fluid to WM voxels. Furthermore, no smoothing is needed. However, abnormalities in the WM might reduce the FA value of a tract locally, and it is possible that the voxel chosen to the skeleton is not actually in the center of the tract. Therefore, we transformed the TBSS results back to native space of each subject to confirm that a given point in the skeleton was derived from the correct anatomically corresponding region. A limitation of TBSS is that by studying only the voxels on the skeleton with the highest FA, a large part of WM remains uninvestigated. The tract-level analysis with the CSD-based tractography method complements the more local TBSS approach, as it is not limited to the voxels with the highest FA in the cross-section of the tract.

### Tract-level analysis with constrained spherical deconvolution tractography

CSD-based tractography showed an increased FA in the right IFO, left ILF, right and left SLF and left UNC in individuals with AS compared to controls, as shown in Table 1, but only the difference in the left ILF was significant after the Bonferroni correction (corrected *P* value = 0.02633). However, Bonferroni correction for multiple comparisons is very conservative and does not account for the correlation between the FA values of different tracts. Thus, in regard to the previous global finding and the results of TBSS, it is possible that true increases in FA may be present in also other tracts than left ILF. No significant changes were found in MD, CP or LI in any of the tracts between the two groups.

**Table 1:** **Fractional anisotropy (FA) values in individuals with Asperger syndrome (AS) and controls in the white matter (WM) tracts reconstructed with tractography**

WM ^a^ tract	FA ^a^ (AS ^a^ subjects) ^b^	FA ^a^ (controls) ^b^	***P***value ^c^	Corrected ***P***value ^c^
**ATR** ^**a**^ **L**	0.3416 ± 0.0381	0.3335 ± 0.0330	0.2629	3.418
**ATR** ^**a**^ **R**	0.3511 ± 0.0399	0.3453 ± 0.0332	0.3330	4.329
**SCC** ^**a**^	0.4521 ± 0.0225	0.4401 ± 0.0553	0.1995	2.593
**CST** ^**a**^ **L**	0.4957 ± 0.0254	0.4848 ± 0.0405	0.1757	2.284
**CST** ^**a**^ **R**	0.4915 ± 0.0278	0.4713 ± 0.0420	0.05339	0.6941
**IFO** ^**a**^ **L**	0.4381 ± 0.0336	0.4129 ± 0.0523	0.05162	0.6710
**IFO** ^**a**^ **R**	0.4365 ± 0.0261	0.4112 ± 0.0342	0.01095	0.1423
**ILF** ^**a**^ **L**	0.3864 ± 0.0277	0.3521 ± 0.0358	0.002026	**0.02633**
**ILF** ^**a**^ **R**	0.3697 ± 0.0258	0.3511 ± 0.0380	0.05233	0.6803
**SLF** ^**a**^ **L**	0.4266 ± 0.0154	0.3978 ± 0.0433	0.006523	0.08480
**SLF** ^**a**^ **R**	0.3899 ± 0.0255	0.3620 ± 0.0434	0.01405	0.1826
**UNC** ^**a**^ **L**	0.3426 ± 0.0368	0.3109 ± 0.0424	0.01470	0.1911
**UNC** ^**a**^ **R**	0.3353 ± 0.0373	0.3143 ± 0.0408	0.06743	0.8766

^a^FA, fractional anisotropy; AS, Asperger syndrome; WM, white matter; ATR, anterior thalamic radiation; SCC, splenium of corpus callosum; CST, corticospinal tract; IFO, inferior fronto-occipital fasciculus; ILF, inferior longitudinal fasciculus; SLF, superior longitudinal fasciculus; UNC, uncinate fasciculus.

^b^Mean and standard deviations are presented for both groups.

^c^Both uncorrected and Bonferroni-corrected *P* values from two-sample t-tests are presented. A significant *P* value (corrected *P* <0.05) is highlighted in bold.

To see if the findings in FA correlated with a specific area such as empathy or systemizing skills, we performed a correlation analysis of AQ, EQ and SQ with FA. Results are shown in Table 2 (no correction for multiple comparison was applied). In the whole sample, a positive correlation of AQ and FA was found in the right IFO. However, the finding could be explained by the highly significant between-group difference in AQ, as no significant correlation was found when the groups were tested separately. A negative correlation of EQ and FA was found in the left ATR, which was supported by a trend of negative correlation in the control subjects (corr = -0.4328, *P* = 0.06417). No correlation was found between SQ and FA in any of the WM tracts.

**Table 2:** **Correlation of autism spectrum quotient (AQ), empathy quotient** (**EQ) and systemizing quotient (SQ) with fractional anisotropy of the white matter tracts**

WM ^a^ tracts	AQ ^a^ and FA ^a^		EQ ^a^ and FA ^a^		SQ ^a^ and FA ^a^	
	Correlation ^b^	***P***value ^c^	Correlation ^b^	***P***value ^c^	Correlation ^b^	***P***value ^c^
**ATR** ^**a**^ **L**	0.3010	0.08871	−0.3551	**0.04255**	0.07749	0.6682
**ATR** ^**a**^ **R**	0.1772	0.3239	−0.2403	0.1779	0.1201	0.5055
**SCC** ^**a**^	0.09482	0.5997	−0.06295	0.7278	0.01877	0.9174
**CST** ^**a**^ **L**	0.1720	0.3385	0.1040	0.5648	−0.004599	0.9797
**CST** ^**a**^ **R**	0.2603	0.1435	0.05810	0.7481	0.07059	0.6963
**IFO** ^**a**^ **L**	0.2675	0.1323	−0.3043	0.08511	0.04044	0.8232
**IFO** ^**a**^ **R**	0.3869	**0.02613**	−0.3213	0.06829	0.1149	0.5242
**ILF** ^**a**^ **L**	0.2861	0.1065	−0.2277	0.2026	0.1736	0.3339
**ILF** ^**a**^ **R**	0.3160	0.07321	−0.2515	0.1580	0.1161	0.5199
**SLF** ^**a**^ **L**	0.3189	0.07049	−0.1911	0.2866	0.1285	0.4759
**SLF** ^**a**^ **R**	0.2730	0.1242	−0.1050	0.5608	0.04742	0.7933
**UNC** ^**a**^ **L**	0.2994	0.09054	−0.07811	0.6657	0.1500	0.4047
**UNC** ^**a**^ **R**	0.1613	0.3700	−0.08445	0.6403	0.09923	0.5827

^a^AQ, Autism spectrum quotient; EQ, Empathy quotient; SQ, Systemizing quotient; WM, white matter; FA, fractional anisotropy; ATR, anterior thalamic radiation; SCC, splenium of corpus callosum; CST, corticospinal tract; IFO, inferior fronto-occipital fasciculus; ILF, inferior longitudinal fasciculus; SLF, superior longitudinal fasciculus; UNC, uncinate fasciculus.

^b^The correlations were calculated for all subjects.

^c^Significant *P* values (*P* <0.05) are written in bold.

### Possible causes for increased fractional anisotropy

Anisotropic diffusion is primarily caused by dense packing of axons and their cell membranes [11]. Other tissue properties such as myelination can also affect the degree of anisotropy [15, 51]. However, one of the most important factors affecting the FA is the complexity of the underlying WM fiber structure [29, 41]: less crossing fiber configurations in individuals with AS could be a cause for the increased FA. However, no between-group differences were observed in CP with either TBSS or with CSD-based tractography. Thus, the higher FA in subjects with AS was not explained by a lower degree of crossing fibers [51]. In addition, it has been recently reported that differences in subject motion may affect DW-MRI metrics in ASD [52, 53]. However, in our sample, there were no differences in subject motion between the groups. Thus, subject motion does not confound our results. FA can increase or decrease without changes in MD. For instance, if axial diffusivity increases and radial diffusivity decreases, FA can increase without changes in MD.

Increased FA indicates that the coherence of the WM tracts in subjects with AS may be higher than in controls [54]. However, it has been suggested that individuals with ASD have difficulties in differentiating signal from noise, and thus, strong physical connectivity does not necessarily equal high computational connectivity [4]. Interestingly, asynchronous brain activity during film watching in individuals with AS was reported in a partly overlapping sample [55]. It is also possible that more intensive training of the social and communication (or other lacking) skills may lead to increased FA values in adults with AS, as in two studies with healthy subjects it has been shown that training can induce an increase in FA [56, 57]. In addition, Pardini and coworkers found an increase in FA in individuals with ASD who highly adhered to therapy, compared to those who adhered to it only moderately [58].

### Comparison to the previous findings

Although most of the previous studies in adults with ASD have reported decreased FA values [18–23], increased FA values have also been found [19, 20]. In addition, a recent study reported a positive correlation between FA and autistic traits in subjects with ADHD most prominent in socio-communicative skills, which are impaired in AS [59]. In some studies in adults with ASD, no group differences in FA related measures were reported [60, 61]. A voxel-based meta-analysis showed increased WM volume in the right arcuate, left inferior fronto-occipital and uncinate fasciculi in individuals with ASD [62]. In studies performed in children and adolescents, more increases in FA have been found than in adults [63–68]. However, the methods and also regions of interest vary a lot between different studies.

CSD-based tractography has been previously used in only two studies in ASD [32, 33]. McGrath and coworkers investigated the WM integrity of the arcuate fasciculus (AF) and IFO, and found a decrease in FA in the right IFO in individuals with ASD [32]. We did not perform tractography of the AF, as it did not appear in the TBSS results, and the FA of the right IFO was increased in our subjects with AS compared to controls in TBSS. At the tract-level, the difference in the right IFO did not endure the correction for multiple comparisons. However, the difference of the absolute values is bigger in our study than in McGrath and coworkers’ study. The samples also differ, as the patients in our study are older (28.6 ± 5.7 versus 17.28 ± 2.87), and have a higher IQ (125.1 ± 14.5 versus 106.84 ± 14.54). McGrath and coworkers also investigated WM microstructure based on the results of functional connectivity and found reduced FA in the WM connecting the left Brodmann area 19 to the left caudate head and to the left thalamus in individuals with ASD [33].

The inconsistent findings in the previous studies may be partly due to the use of different methods, but also to the heterogeneity of the samples, as in ASD, the diversity of symptoms is large and variation in the degree of their severity among individuals varies a lot. Differences in age and IQ between the different samples may also affect the results, as it has been shown that FA values correlate with IQ [69] and that FA changes with age [53, 70]. Langen and coworkers have hypothesized that there might be an earlier peak in the WM maturation and an earlier onset of an age-related decrease in FA in autism [22]. In our study, we have chosen a homogeneous sample of subjects with ASD, as subjects with AS do not have a clinically significant delay in speech and cognitive development (International Classification of Disease; World Health Organization; 1993). In addition, our subjects with AS are high-functioning, with a mean IQ of 125.1 ± 14.5.

### Advantages of the chosen methodology

The large amount of different analysis methods makes it more difficult to compare different studies with each other, as each of the methods has its limitations [24, 25, 71, 72]. Because of the inconsistency in the results of the previous studies, we have used two different techniques to reliably detect the possible WM changes in male adults with AS. In TBSS, the FA of the WM is investigated at a more local level than in the tractography-based analysis, where the mean FA is calculated for the whole tract. Therefore, possible differences in some part of the WM tract seen in TBSS may not be seen in the mean FA of the whole tract. In that sense, the TBSS analysis might be more sensitive, but the tractography-based analysis might be more robust. Furthermore, in the tractography approach we have used CSD-based tractography instead of DTI-based tractography. Unlike in DTI, with CSD it is possible to reliably investigate crossing fibers, which are present in up to 90% of the WM tissue [29].

Thus, TBSS and CSD-based tractography complement each other, and both indicate increased FA in individuals with AS. Furthermore, these results are supported by our earlier findings of globally increased FA in subjects with AS [27], but provide more information concerning the location of the differences.

### Limitations

Limitations of our study include a relatively small sample size. However, our results survived corrections for multiple comparisons and thus, can be considered robust. Finland is an isolated and genetically homogeneous country [73], which can be beneficial as it has been suggested that heritable factors play a strong role in WM organization [69]. Another limitation concerns the Autism Diagnostic Interview-Revised (ADI-R) and Autism Diagnostic Observation Schedule (ADOS). They are standard instruments in the diagnostics of ASD in many countries, but were not in use in Finland at the time of the study. Therefore, we do not have this information for our subjects. However, we used AQ, EQ and SQ questionnaires specifically designed for high-functioning individuals with ASD, and in addition, all subjects were thoroughly screened to exclude other psychiatric disorders. In addition, the DW-MRI acquisition was suboptimal for CSD-based tractography [48], as the diffusion-weighting was relatively low and the voxel size was anisotropic. Nevertheless, fiber crossings, present in 60 to 90% of the WM voxels [29], can reliably be identified with CSD [48, 74, 75], and thus, using CSD is highly beneficial in comparison to DTI, where fiber crossings cannot be identified. Finally, we used a semi-automated tract segmentation method to extract the 13 fiber tracts in 33 subjects [50]. While this approach has been successfully applied for DTI-based tractography using a single representative subject [50, 76], performance characteristics for CSD-based tractography, combined with the use of more advanced population-specific atlas templates [77, 78], remain to be determined.

## Conclusions

We investigated local microstructural differences in the WM of individuals with AS by comparing their FA, MD and CP values to those of age-, sex- and IQ-matched controls. We used a dual approach and performed first a more local voxelwise analysis, TBSS, and then confirmed the findings with a tract-level analysis approach, CSD-based tractography. Our results suggest that there are widely distributed abnormalities in the WM tracts of adults with AS, and that these are most pronounced in the left ILF. The changes were not explained by differences in the complexity of the microstructural organization of the WM. Furthermore, these results are in line with our earlier findings of globally increased FA in subjects with AS [27].

Our study was restricted to adult males with AS. In the future, longitudinal studies beginning in the childhood and following the diagnosis and possible rehabilitation should be performed to more thoroughly understand the neural deficits and the effects of age and IQ in ASD. Furthermore, as ASD is a very broad and heterogeneous disorder, the study samples should be as homogeneous as possible, and different symptom domains should be investigated separately. Finally, the WM tracts could be segmented and thus, short parts of the tracts could be investigated separately in addition to looking at the mean FA of the whole tract.

## Authors’ information

UR has two academic degrees, a Master of Science (M.Sc.) in Technology from Aalto University (2010) and medical degree from the University of Helsinki (2011), acquired simultaneously. In the beginning of her medical studies she was chosen to a graduate program for medical doctors, and she will soon finish her doctoral studies, during which she has attended multiple scientific conferences and courses and visited research groups abroad. She’s especially interested in neuropsychiatry, diffusion magnetic resonance imaging and applying the newest technology to clinical studies. JS is a researcher and a psychologist. He has a PhD in psychology. He is currently working as a clinical psychologist helping children and adults with neurodevelopmental disabilities and as a post-doctoral researcher in the field of cognitive neuroscience. TR received his M.Sc. (Tech.) in 2009 from Helsinki University of Technology (currently Aalto University), Finland. He did research on mineral processing technology in the Control Engineering group of Aalto University from 2005 to 2010. From 2010 to 2012 he worked as a consultant for healthcare management in Nordic Healthcare Group, Finland. In 2012 he started his PhD about diffusion MRI and constrained spherical deconvolution at the iMinds-Vision Lab, Department of Physics, University of Antwerp, Belgium under the supervision of Ben Jeurissen, Alexander Leemans and Jan Sijbers. TNvW is a pediatrician and a child neurologist. She did her PhD on Asperger syndrome, and she is a specialist in diagnostics, neuroimaging and molecular genetics of AS. TNvW has a wide experience in neuropsychiatric diagnostics, and she treats both children and adults with AS. She is the managing director of the Neuropsychiatric Rehabilitation and Medical Centre Neuromental in Helsinki, Finland. SL is an adjunct professor of psychiatry at the University of Helsinki. He has ten years of experience with developmental neuropsychiatric disorders and is especially interested in clinical manifestations of ADHD and ASD in adulthood. In addition, he has done research on mood disorders and chronobiology. PR is an MD and a PhD. He has two specialties regarding neuropsychiatric disorders: he is a pediatric neurologist and an adolescent psychiatrist. In addition, he has five years of brain imaging experience with neuropsychiatric disorders at UCLA PET-Center and Neuropsychiatric Center. In Finland, he has clinical work experience with neuropsychiatric disorders including Asperger syndrome and other ASD, and he works at the Helsinki University Central Hospital Adolescent Psychiatry Clinic. His main field is evaluating neuropsychiatric patients and their comorbid conditions in addition to medical treatment and rehabilitation. PT is an adjunct professor of psychiatry at the University of Helsinki. He has more than ten years of experience on developmental neuropsychiatric disorders and is especially interested in clinical manifestation of ASD in adulthood. In addition, he has done research in forensic psychiatry. AL is a physicist who received his PhD in 2006 at the University of Antwerp, Belgium. From 2007 to 2009, he worked as a postdoctoral researcher at the Cardiff University Brain Research Imaging Center (CUBRIC), Cardiff University, Wales, United Kingdom. In 2009, he joined the Image Sciences Institute (ISI), University Medical Center Utrecht, the Netherlands, where he currently holds a tenured faculty position as Associate Professor. His current research interests include modeling, processing, visualizing and analyzing diffusion MRI data for investigating microstructural and architectural tissue organization. He heads the PROVIDI Lab and is the developer of the software ExploreDTI. MS is a professor of cognitive neuroscience in Aalto University. He is very experienced in noninvasive study of neural basis of cognitive functions such as attention, speech perception, auditory perception and multisensory integration. Now his research is focused on studying brain activity in naturalistic conditions.

## Abbreviations

ADI-R: Autism Diagnostic Interview–revised
ADOS: Autism Diagnostic Observation Schedule
AF: arcuate fasciculus
AQ: autism spectrum quotient
AS: Asperger syndrome
ASD: autism spectrum disorder
ATR: anterior thalamic radiation
CP: planar diffusion coefficient
CSD: constrained spherical deconvolution
CST: corticospinal tract
DTI: diffusion tensor imaging
DW: diffusion weighted
EQ: empathy quotient
Eyes Test: Reading the Mind in the Eyes Test
FA: fractional anisotropy
FDT: FMRIB’s diffusion toolbox
FMRIB: Functional MRI of the Brain
FOD: fiber orientation distribution
FOV: field of view
FRT: Benton Facial Recognition Test
FSL: FMRIB software library
GM: gray matter
IFO: inferior fronto-occipital fasciculus
ILF: inferior longitudinal fasciculus
LI: laterality index
MD: mean diffusivity
MRI: magnetic resonance imaging
NEX: number of excitations
PTR: posterior thalamic radiation
PVE: partial volume effect
SCC: splenium of corpus callosum
SLF: superior longitudinal fasciculus
SQ: systemizing quotient
TBSS: tract-based spatial statistics
TE: echo time
TFCE: threshold-free cluster enhancement
TR: repetition time
UNC: uncinate fasciculus
WM: white matter.
